# Unraveling the indolence of papillary thyroid carcinoma: an exploratory study on B-cell subsets based on genetic predisposition and tumor immunity

**DOI:** 10.3389/fimmu.2026.1769020

**Published:** 2026-03-18

**Authors:** Pei Wang, Zhizhong Dong, Xing Peng, Cong Zhou, Ruochuan Cheng, Wen Liu

**Affiliations:** 1 Department of Radiation Oncology, Cancer Institute, The First Affiliated Hospital and College of Clinical Medicine of Henan University of Science and Technology, Luoyang, China; 2 Department of Thyroid Surgery, Clinical Research Center for Thyroid Disease of Yunnan Province, The First Affiliated Hospital of Kunming Medical University, Kunming, China; 3 School of Mental Health, Jining Medical University, Jining, China

**Keywords:** active surveillance, B cell, indolent, Mendelian randomization, papillary thyroid carcinoma

## Abstract

**Background:**

Active surveillance for low-risk papillary thyroid carcinoma (PTC) is hampered by the lack of reliable biomarkers to distinguish indolent from progressive tumors. While our previous single-cell analysis identified tumor-infiltrating B cells as key determinants of indolent PTC, their clinical utility remains constrained by low abundance and peripheral undetectability. We therefore employed Mendelian randomization (MR) to investigate this causal relationship and assess the potential of peripheral B-cell profiling as a non-invasive strategy for distinguishing indolent PTC.

**Methods:**

We integrated MR, flow cytometry, single-cell transcriptomics, and clinical validation. A two-sample MR framework was used to assessed causal relationships between immunophenotypes and thyroid cancer risk. Findings were exploratorily investigated by flow cytometric comparison of B-cell subsets between indolent and progressive PTC patients, further characterized using single-cell RNA sequencing data, and evaluated for prognostic significance in the TCGA-THCA cohort.

**Results:**

Multivariable MR identified CD20+ IgD+ CD38^-^ naïve B cells, CD27+ unswitched memory B cells and CD3 on activated CD4 regulatory T cells as independent protective factors thyroid cancer susceptibility (OR<1, *P* < 0.05), supporting a potential link between thyroid cancer-susceptible B-cell phenotypes and indolent tumor behavior. Flow cytometry confirmed a significantly higher proportion of peripheral naïve B-cell in indolent compared progressive PTC (70.8% vs. 60.9%, *P* = 0.032). scRNA-seq revealed these subsets as the predominant tumor-infiltrating B populations in indolent PTC. In the TCGA cohort, high enrichment scores for these B-cell subsets were associated with improved T stage. Furthermore, among patients ≥55 years, high naïve B-cell scores correlated with improved disease-free survival (DFS) (HR = 0.233, *P* < 0.001) and overall survival (OS) (HR = 0.292, *P* = 0.0111), while high CD27+ memory B-cell levels were associated with better DFS (HR = 0.212, *P* < 0.001) and OS (HR = 0.346, *P* = 0.0326).

**Conclusion:**

This study provides exploratory genetic and clinical evidence supporting a causal, protective role for specific peripheral and tumor-infiltrating B-cell subsets in PTC. Naïve B cells and CD27+ unswitched memory B cells are linked to indolent tumor behavior and favorable prognosis, highlighting their potential as biomarkers for risk stratification and non-invasive monitoring in PTC management.

## 1 Introduction

Global thyroid cancer incidence has risen substantially in recent decades, yet this increase has not been matched by a corresponding decline in mortality. This disparity underscores persistent limitations in contemporary diagnostic and therapeutic paradigms (1, 2). Papillary thyroid carcinoma (PTC), which constitutes approximately 84% of all thyroid cancers, presents a specific clinical challenge (3). In developed regions, low-risk PTCs—typically staged as T1N0M0 and associated with a disease-specific mortality below 1%—are frequently subject to over-diagnosis and overtreatment (4, 5). Active surveillance (AS) has therefore emerged as a viable management strategy for such patients (6). However, its widespread clinical implementation is critically constrained by the absence of reliable biomarkers that can accurately differentiate indolent from progressive tumors at the time of initial diagnosis.

To address this need for objective stratification, clear and quantifiable definitions of tumor behavior are essential. In this study, as in clinical practice, we define indolent PTC​ by a tumor volume doubling rate (TDR) of ≤ 0 per year, indicating stability or regression as assessed through serial ultrasound evaluations. Progressive PTC, conversely, is characterized either by a TDR > 0.5 per year during surveillance or by the development of adverse clinical features such as significant tumor enlargement, lymph node metastasis, or extrathyroidal extension (7, 8). These dual criteria, integrating dynamic growth kinetics with recognized clinical framework, establish a robust framework for consistent patient classification and ensures that subsequent mechanistic comparisons are grounded in clinically meaningful phenotypes.

Our previous single-cell RNA sequencing (scRNA-seq) study identified tumor-infiltrating B cells (TIL-Bs), particularly germinal center B cells (GC-Bs) involved in tertiary lymphoid structure (TLS) formation, as pivotal components of the indolent PTC tumor microenvironment (9). Functional validation confirmed that TIL-Bs can directly suppress PTC cell proliferation in a concentration-dependent manner. Although GC-Bs and related markers hold diagnostic promise for indolent disease, their clinical translation is hampered by low abundance within tumors and undetectability in peripheral blood. Interestingly, we found that indolent PTC tumors possess a superior capacity to recruit B cells from the circulation, a process governed predominantly by tumor-secreted factors rather than B-cell homing receptors. This observation suggests that the protective immune microenvironment in indolent PTC may be shaped by specific circulating B-cell subsets, highlighting a potential avenue for non-invasive, dynamic monitoring.

Despite these compelling correlations, a fundamental question remains unresolved: Do specific B-cell subsets play a causal role in restraining PTC initiation and progression, or are they merely passive passengers that accumulate in indolent, slow-growing tumors? To move beyond association and investigate causality in disease susceptibility, we employed Mendelian Randomization (MR), a methodological approach that uses genetic variants as instrumental variables to minimize confounding and infer causal relationships (10, 11).

Building on the correlative findings from our prior scRNA-seq research, we therefore hypothesize—as an exploratory step—that the abundance of specific peripheral blood B-cell subsets exerts a causal influence on PTC development. This exploratory study integrates MR analysis, flow cytometry, single-cell transcriptomics, and clinical validation to preliminarily interrogate causal directionality and identify potential protective B-cell subsets. Our aim is to clarify promising research pathways towards developing biomarkers for improved risk stratification and non-invasive monitoring in PTC management.

## 2 Materials and methods

### 2.1 Human tumor specimens

As previously outlined, we conducted a prospective observation cohort with AS management for patients with low-risk PTC (12, 13). Patients underwent ultrasound monitoring at 6- to 12-month intervals. In this study, “indolent PTC” is objectively defined as papillary thyroid carcinoma exhibiting a tumor volume doubling rate (TDR) ≤ 0/year during AS, indicating no significant growth over a minimum of three serial ultrasound assessments. In contrast, “progressive PTC” is defined by either a TDR > 0.5/year during surveillance or the presence of clinically evident disease at presentation, such as a palpable mass or confirmed lymph node metastasis (N1 stage).

### 2.2 Two-sample mendelian randomization study

#### 2.2.1 Data sources and preparation

The exposure factors (immunophenotypes) as well as outcome variable (thyroid cancer, TC) were downloaded from the integrative epidemiology unit (IEU) Open genome-wide association study (GWAS) database (https://gwas.mrcieu.ac.uk/). Minutely, genetic variation data for 731 peripheral blood immunophenotypes were sourced from the largest GWAS to date on immune phenotypes (GWAS Catalog registration numbers GCST0001391-GCST0002121) (14) (**Supplementary Table 1**). The TC-related dataset (ebi-a-GCST90018929) from the IEU OpenGWAS database, which included 24,198,226 single nucleotide polymorphisms (SNPs) from 491,974 European samples (cases: controls = 1,054: 490,920).

#### 2.2.2 Instrumental variables screening

The exposure factors were initially read and independent SNPs associated with exposure factors were identified by the extract_instruments function of the TwoSampleMR package (v 0.5.6). To minimize weak instrument bias and ensure the reliability of causal inference, the genome-wide significant p-value < 5×10– (6) was adopted as the core screening criterion, combined with F-statistic > 10 to further exclude weak instruments with low explanatory power for exposure factors. To circumvent potential bias resulting from linkage disequilibrium (LD), LD pruning was performed with parameters: clump = TRUE, r² = 0.001, kb = 10,000 to eliminate overlapping SNPs and reduce multicollinearity. Subsequently, the extract_outcome_data function was used to obtain the IVs (SNPs) corresponding to the exposure factors in the outcome dataset (proxies = TRUE, rsq = 0.8). Afterwards, the harmonise_data function was employed to unify the effect alleles and effect sizes, and SNPs linked to TC were further excluded to avoid causal interference. Following the above stringent screening criteria, 637 immunophenotypes were retained from the original 731, and the data on IVs related to the cell sub-types, including T lymphocytes (n = 310), B lymphocytes (n = 192), myeloid cells (n = 38), monocytes (n = 64), and natural killer (NK) cells (n = 33), are summarized in **Supplementary Table 2**.

#### 2.2.3 Two-sample Mendelian randomization and sensitivity analysis

Five algorithms (MR Egger) (15), weighted median (16), inverse variance weighted (IVW) (17), unweighted regression (18), maximum likelihood (19)were included to analyze the relationship between exposure factors and TC via MR function, and IVW was the main method. If the IVW method was significant (p-value < 0.05), a causal relationship was deemed to exist between the exposure factors and TC. Particularly, the Forest plot package (v 2.0.1) (20)was used to plot forest plots to determine the causal effects of each SNP on TC. Scatter plots were drawn based on the IVW method to ascertain the correlation between exposure factors and TC. Funnel plots were used to assess the potential for horizontal pleiotropy by evaluating plot symmetry, a key indicator for detecting pleiotropic bias in Mendelian randomization analysis.

Sensitivity analyses were conducted to validate the robustness of causal inferences using the TwoSampleMR package and MR-PRESSO package, including: (1) Heterogeneity test: Cochran’s Q test (MR-IVW and MR-Egger methods), with p-value > 0.05 indicating no significant heterogeneity; (2) Horizontal pleiotropy test: MR-Egger intercept test and MR-PLEIOTROPY RESIDUAL SUM AND OUTLIER (MR-PRESSO) global test, with p-value > 0.05 considered as no significant horizontal pleiotropy. The MR-PRESSO outlier test was further performed to identify and remove pleiotropic outlier SNPs, and the corrected causal effect was recalculated; (3) Leave-one-out (LOO) test: to verify whether individual SNPs drive the overall causal results; (4) Steiger’s directionality test: performed by the directionality_test function (correct causal direction = TRUE, p-value < 0.05) to confirm the correct causal direction between exposure and outcome. Additionally, Mendelian randomization robust adjusted profile score (MR-RAPS) was performed as an additional sensitivity analysis to further address the potential impact of weak instruments and unmeasured confounding; MR-RAPS downweights the effects of weak instruments and pleiotropic SNPs, and the consistency of its results with the main IVW results was used to validate the robustness of causal inferences.

#### 2.2.4 Multivariate MR analysis and reversal MR test

To identify immune cell types exhibiting independent causal relationships with TC, we performed multivariate MR analysis. Instrumental variables were selected using the same criteria as described for the primary MR analysis. Collinear variables were excluded using a LASSO-based feature selection approach. The analysis was implemented via the mv multiple function to pinpoint key immune factors significantly associated with TC (*P* < 0.05).

Subsequently, reversal MR analysis was performed utilizing the TwoSampleMR package, with TC (ebi-a-GCST90018929) as the exposure factor and the key immune factors as the outcomes, following the same analytical procedures as the primary MR framework. Instrumental variables were selected using the same stringent criteria (*P* < 5×10^-6^, F-statistic>10, LD pruning) as described for the primary MR analysis.

### 2.3 Flow cytometry

Peripheral blood samples were collected preoperatively from 5 patients with indolent PTC and 5 with progressive PTC (**Supplementary Table 3**). Peripheral blood mononuclear cells (PBMCs) were isolated by density gradient centrifugation and counted using a Countstar automated cell counter. Cells were then incubated with 5 μL Fc receptor blocking reagent and Zombie NIR viability dye (BioLegend, USA) for 15 minutes at room temperature in the dark. Following blocking, cells were stained with a surface antibody cocktail (BioLegend, USA) containing CD45-PE, CD20-FITC, IgD-PE/Cyanine7, and CD27-APC for 30 minutes under light-protected conditions. After staining, cells were washed once with Cell Staining Buffer, centrifuged at 500 × g for 5 minutes at 4 °C, and resuspended in 400 μL PBS for acquisition. Samples were analyzed on a NovoCyte Opteon spectral flow cytometer (Agilent Technologies, USA), and data processing was performed using FlowJo software (Tree Star, v10.8.1, USA).

To justify the sample size and ensure sufficient statistical power, a power analysis was performed using G*Power 3.1.9.7 software. Based on the large effect size (d=1.8) of naïve B cell proportion differences observed in our preliminary experiment, with a two-tailed α=0.05 and desired power (1-β)=0.8, the results showed that the minimum sample size required per group was 4. The actual sample size in this study (n=5 per group) meets the statistical power requirement, ensuring the reliability of detecting intergroup differences in B-cell subset proportions.

### 2.4 Analysis of bulk RNA-seq data

We analyzed bulk RNA sequencing (RNA-seq) data from patients with papillary thyroid carcinoma (PTC) obtained from The Cancer Genome Atlas (TCGA). To minimize the potential confounding influence of autoimmune thyroid diseases (such as Hashimoto’s thyroiditis and Graves’ disease) on the tumor immune microenvironment, we strictly excluded patients with these comorbidities. Using gene sets specifically highly expressed in relevant B-cell subsets (**Supplementary Table 4**), as identified from our single-cell transcriptomic data, we further evaluated their associations with overall survival (OS) and disease-free survival (DFS).

Differentially expressed genes (DEGs) between B cell subsets in indolent and progressive PTC were identified using the Seurat package (v4.3.0) with the criteria of |log2FC| > 1 and adjusted P < 0.05. Gene Ontology (GO) and Kyoto Encyclopedia of Genes and Genomes (KEGG) enrichment analyses were performed using the clusterProfiler package (v4.6.0) to annotate the biological functions of DEGs, with a focus on immune-related pathways such as antigen processing and presentation, cytokine-cytokine receptor interaction, and lymphoid structure formation.

### 2.5 Tumor microenvironment analysis

To align the genetic causal inferences from MR with histological evidence, we integrated and re-analyzed previously published single-cell RNA sequencing (scRNA-seq) data (9). Cell-state transitions and differentiation trajectories were reconstructed using the Monocle2 package (v2.14.0). We further leveraged bulk RNA-seq data from PTC patients in the TCGA database. Gene sets representative of key B-cell populations were derived, and enrichment scores for each patient were calculated using the Xcell algorithm. These scores were compared across PTC tumors of different T stages. Differential expression analysis of individual genes was performed using the Stat_compare_means function from the R package ggpubr. For the B-cell subset enrichment scores calculated by the Xcell algorithm, patients were stratified into high and low enrichment score groups by the cut point of the corresponding scores, and the low enrichment score group was set as the reference group (Ref=1) for subsequent survival analysis to assess the prognostic value of B-cell infiltration in PTC.

### 2.6 Ethics

This study utilized publicly available GWAS data (ethically approved by their original studies) and prospectively collected patient samples. All prospective experiments, including flow cytometry and scRNA-seq, were approved by the Ethics Committee of the First Affiliated Hospital of Kunming Medical University, with informed consent obtained from all participants.

### 2.7 Statistical analysis

All statistical analyses were performed using R software (version 4.3.1), SPSS software (version 26.0) and GraphPad Prism (version 9.0). GraphPad Prism and R package ggplot2 were used for data visualization and figure generation. A two-sided P < 0.05 was considered statistically significant for all analyses unless otherwise specified.

## 3 Results

### 3.1 Genetic evidence for B-cell-mediated protection in thyroid cancer

#### 3.1.1 Initial MR screening identifies significant immunophenotypes

Through a series of stringent screening criteria, we ultimately obtained 2,854 independent SNPs as IVs (**Supplementary Table 5**). Two-sample MR analysis based on the IVW method was performed, and false discovery rate (FDR) correction was applied for multiple comparisons of 731 immune phenotypes, and the following identified associations are defined as exploratory findings for hypothesis generation; FDR correction was additionally applied for multiple comparisons as a supplementary validation. After FDR correction (q-value < 0.05), 17 immune exposure factors were still significantly associated with thyroid cancer susceptibility (**Table 1**). Among them, 4 were risk factors (OR > 1), including ebi-a-GCST90001784 (CD25 on IgD+ CD38dim B cells) and ebi-a-GCST90001794 (CD25 on IgD+ B cells), etc.; the remaining 13 were protective factors (OR < 1), indicating that the exploratory significant associations were not caused by false positives from multiple hypothesis testing.

**Table 1:** Causal effects of 17 immune-related exposure factors on thyroid cancer identified by the inverse variance weighted method.

Outcome	Exposure	Method	nsnp	pval	or	or_lci95	or_uci95
Thyroid cancer || id:ebi-a-GCST90018929	|| id:ebi-a-GCST90001749	Inverse variance weighted	11	0.036930039	0.867834747	0.759639458	0.991440268
Thyroid cancer || id:ebi-a-GCST90018929	|| id:ebi-a-GCST90001784	Inverse variance weighted	18	0.005431539	1.123248859	1.03488461	1.219158142
Thyroid cancer || id:ebi-a-GCST90018929	|| id:ebi-a-GCST90001787	Inverse variance weighted	13	0.013889557	0.899531811	0.826762713	0.97870582
Thyroid cancer || id:ebi-a-GCST90018929	|| id:ebi-a-GCST90001794	Inverse variance weighted	19	0.024457951	1.073263505	1.009150692	1.141449497
Thyroid cancer || id:ebi-a-GCST90018929	|| id:ebi-a-GCST90001806	Inverse variance weighted	25	0.03375762	0.896850199	1.012834058	1.285691108
Thyroid cancer || id:ebi-a-GCST90018929	|| id:ebi-a-GCST90001813	Inverse variance weighted	16	0.030040155	1.141136163	1.012834058	1.285691108
Thyroid cancer || id:ebi-a-GCST90018929	|| id:ebi-a-GCST90001456	Inverse variance weighted	11	0.046820397	0.809393112	0.780331186	1.058165843
Thyroid cancer || id:ebi-a-GCST90018929	|| id:ebi-a-GCST90001479	Inverse variance weighted	10	0.049606453	0.86138317	0.815116022	1.124011984
Thyroid cancer || id:ebi-a-GCST90018929	|| id:ebi-a-GCST90001615	Inverse variance weighted	14	0.040893356	0.854007615	0.775500951	1.045133029
Thyroid cancer || id:ebi-a-GCST90018929	|| id:ebi-a-GCST90001842	Inverse variance weighted	22	0.040974337	0.921586402	0.979510485	1.521964124
Thyroid cancer || id:ebi-a-GCST90018929	|| id:ebi-a-GCST90001853	Inverse variance weighted	13	0.040906371	0.904598658	0.597192452	0.979509248
Thyroid cancer || id:ebi-a-GCST90018929	|| id:ebi-a-GCST90001871	Inverse variance weighted	14	0.002488094	0.867379884	0.847500641	1.00575907
Thyroid cancer || id:ebi-a-GCST90018929	|| id:ebi-a-GCST90002002	Inverse variance weighted	10	0.031017728	0.833001079	0.755788473	1.120745681
Thyroid cancer || id:ebi-a-GCST90018929	|| id:ebi-a-GCST90002011	Inverse variance weighted	8	0.034097615	0.790221216	0.777900984	1.196187982
Thyroid cancer || id:ebi-a-GCST90018929	|| id:ebi-a-GCST90002058	Inverse variance weighted	16	0.013261944	0.849625101	0.81529055	1.052752869
Thyroid cancer || id:ebi-a-GCST90018929	|| id:ebi-a-GCST90002068	Inverse variance weighted	16	0.016419789	0.882177721	0.819838529	1.032413674
Thyroid cancer || id:ebi-a-GCST90018929	|| id:ebi-a-GCST90002085	Inverse variance weighted	14	0.030904483	1.168012839	0.978115655	1.289182087

Scatter plots showed that positive slopes based on the IVW method represented risk factors, while negative slopes represented protective factors (**Supplementary Figure 1**). Forest plots visually displayed the effect size of each exposure factor (**Supplementary Figure 2**), and the symmetrical distribution of SNPs in funnel plots indicated the absence of significant horizontal pleiotropic bias in the MR analysis, further validating the reliability of the causal inference results (**Supplementary Figure 3**).

#### 3.1.2 Multivariable MR analysis further pinpoints three core B-cell protective factors

To further refine the exploratory immune phenotypes identified in the initial MR screening and identify independent causal immune cell subsets with thyroid cancer susceptibility, we performed Multivariable MR analysis. The results indicated that the following three B-cell subsets were significant protective factors for TC (OR < 1, *P* < 0.05), defined as key immune factors (**Table 2**):

**Table 2:** Results of multivariable Mendelian randomization analysis identifying key immune factors associated with thyroid cancer.

Id.exposure	Exposure	Id.outcome	Outcome	nsnp	pval	or	or_lci95	or_uci95
ebi-a-GCST90001749	CD20 on IgD+ CD38- naive B cell || id:ebi-a-GCST90001749	ebi-a-GCST90018929	Thyroid cancer || id:ebi-a-GCST90018929	0	0.00982005	0.84588155	0.7449589	0.96047662
ebi-a-GCST90001784	CD25 on IgD+ CD38dim B cell || id:ebi-a-GCST90001784	ebi-a-GCST90018929	Thyroid cancer || id:ebi-a-GCST90018929	3	0.13933275	1.5273702	0.87108195	2.67811741
ebi-a-GCST90001787	CD25 on IgD- CD38- B cell || id:ebi-a-GCST90001787	ebi-a-GCST90018929	Thyroid cancer || id:ebi-a-GCST90018929	3	0.05907516	0.86768347	0.74878924	1.00545595
ebi-a-GCST90001794	CD25 on IgD+ B cell || id:ebi-a-GCST90001794	ebi-a-GCST90018929	Thyroid cancer || id:ebi-a-GCST90018929	2	0.54177753	0.84091881	0.48198182	1.46715999
ebi-a-GCST90001806	CD27 on unswitched memory B cell || id:ebi-a-GCST90001806	ebi-a-GCST90018929	Thyroid cancer || id:ebi-a-GCST90018929	11	0.01455812	0.89290046	0.81533438	0.9778457
ebi-a-GCST90001813	CD38 on IgD+ CD38dim B cell || id:ebi-a-GCST90001813	ebi-a-GCST90018929	Thyroid cancer || id:ebi-a-GCST90018929	4	0.06777708	1.11516868	0.99206348	1.25355001
ebi-a-GCST90001456	CD11c+ HLA DR++ monocyte Absolute Count || id:ebi-a-GCST90001456	ebi-a-GCST90018929	Thyroid cancer || id:ebi-a-GCST90018929	0	0.21782095	0.90869126	0.78033119	1.05816584
ebi-a-GCST90001479	CD4 regulatory T cell %T cell || id:ebi-a-GCST90001479	ebi-a-GCST90018929	Thyroid cancer || id:ebi-a-GCST90018929	1	0.5934488	0.95718346	0.81511602	1.12401198
ebi-a-GCST90001615	TCRgd T cell Absolute Count || id:ebi-a-GCST90001615	ebi-a-GCST90018929	Thyroid cancer || id:ebi-a-GCST90018929	1	0.1675648	0.90027866	0.77550095	1.04513303
ebi-a-GCST90001842	CD3 on Naive CD4+ T cell || id:ebi-a-GCST90001842	ebi-a-GCST90018929	Thyroid cancer || id:ebi-a-GCST90018929	2	0.07575671	1.22097495	0.97951049	1.52196412
ebi-a-GCST90001853	CD3 on activated CD4 regulatory T cell || id:ebi-a-GCST90001853	ebi-a-GCST90018929	Thyroid cancer || id:ebi-a-GCST90018929	0	0.03366864	0.76482386	0.59719245	0.97950925
ebi-a-GCST90001871	HVEM on T cell || id:ebi-a-GCST90001871	ebi-a-GCST90018929	Thyroid cancer || id:ebi-a-GCST90018929	1	0.06747207	0.92324507	0.84750064	1.00575907
ebi-a-GCST90002002	PDL-1 on monocyte || id:ebi-a-GCST90002002	ebi-a-GCST90018929	Thyroid cancer || id:ebi-a-GCST90018929	0	0.4089122	0.92035138	0.75578847	1.12074568
ebi-a-GCST90002011	CD64 on CD14+ CD16+ monocyte || id:ebi-a-GCST90002011	ebi-a-GCST90018929	Thyroid cancer || id:ebi-a-GCST90018929	2	0.74288576	0.96463247	0.77790098	1.19618798
ebi-a-GCST90002058	CD8 on CD8+ T cell || id:ebi-a-GCST90002058	ebi-a-GCST90018929	Thyroid cancer || id:ebi-a-GCST90018929	2	0.2413438	0.92644453	0.81529055	1.05275287
ebi-a-GCST90002068	CD4 on secreting CD4 regulatory T cell || id:ebi-a-GCST90002068	ebi-a-GCST90018929	Thyroid cancer || id:ebi-a-GCST90018929	1	0.15630368	0.9200068	0.81983853	1.03241367
ebi-a-GCST90002085	SSC-A on HLA DR+ CD4+ T cell || id:ebi-a-GCST90002085	ebi-a-GCST90018929	Thyroid cancer || id:ebi-a-GCST90018929	0	0.09978818	1.12292884	0.97811565	1.28918209

**ebi-a-GCST90001749**: CD20 on IgD+ CD38- naïve B cells.
**ebi-a-GCST90001806**: CD27 on unswitched memory B cells.
**ebi-a-GCST90001853**: CD3 on activated CD4 regulatory T cells.

Notably, the first two B-cell subsets align with the predominant populations observed in our prior scRNA-seq analysis of PTC tumors.

#### 3.1.3 Validate the robustness and directionality of causal relationships

Sensitivity analyses further confirmed the accuracy and robustness of the two-sample MR results regarding thyroid cancer susceptibility. Cochran’s Q test showed no significant heterogeneity for all 17 immune exposure factors (*P* > 0.05) (**Supplementary Table 6**), indicating no inter-SNP heterogeneity in causal effects. For horizontal pleiotropy, MR-Egger intercept test showed no significant pleiotropy (*P* > 0.05), and MR-PRESSO global test showed p = 0.42 > 0.05 (**Supplementary Table 7**), indicating no significant horizontal pleiotropy among the selected IVs; no pleiotropic outlier SNPs were identified by the MR-PRESSO outlier test, further confirming that the causal inference results were not affected by residual confounding from pleiotropic effects. LOO analysis showed that removing any single SNP did not significantly alter the overall causal effect estimates (**Supplementary Figure 4**), validating the stability of the results. Steiger directionality tests confirmed the correct causal direction for all 17 exposure factors with TC (*P* < 0.05) (**Supplementary Table 8**). Furthermore, MR-RAPS sensitivity analysis was performed, and the results were fully consistent with the main IVW analysis: the three core protective B-cell subsets still showed a significant causal protective effect on thyroid cancer (OR < 1, *P* < 0.05), which further ruled out the potential impact of weak instruments and unmeasured confounding on the causal inference results. Finally, reverse MR analysis showed that when TC was used as the exposure and the key immune factors as outcomes, all p-values were greater than 0.05 (**Table 3**), ruling out reverse causality and further supporting the unidirectional causal relationship between the identified B-cell subsets and thyroid cancer susceptibility.

**Table 3:** Results of reverse Mendelian randomization analysis between thyroid cancer and the three key immune factors.

Outcome	Exposure	Method	nsnp	b	se	pval
CD20 on IgD+ CD38- naive B cell || id:ebi-a-GCST90001749	|| id:ebi-a-GCST90018929	MR Egger	14	-0.066598302	0.07447142	0.3887566
CD20 on IgD+ CD38- naive B cell || id:ebi-a-GCST90001749	|| id:ebi-a-GCST90018929	Weighted median	14	0.028612443	0.05025544	0.56912529
CD20 on IgD+ CD38- naive B cell || id:ebi-a-GCST90001749	|| id:ebi-a-GCST90018929	Inverse variance weighted	14	0.000847367	0.035612	0.98101658
CD20 on IgD+ CD38- naive B cell || id:ebi-a-GCST90001749	|| id:ebi-a-GCST90018929	Simple mode	14	0.027757477	0.07459877	0.71582064
CD20 on IgD+ CD38- naive B cell || id:ebi-a-GCST90001749	|| id:ebi-a-GCST90018929	Weighted mode	14	0.015365714	0.05733929	0.79292057
CD27 on unswitched memory B cell || id:ebi-a-GCST90001806	|| id:ebi-a-GCST90018929	MR Egger	14	0.035475892	0.05394597	0.52319763
CD27 on unswitched memory B cell || id:ebi-a-GCST90001806	|| id:ebi-a-GCST90018929	Weighted median	14	0.048478522	0.03404984	0.15451827
CD27 on unswitched memory B cell || id:ebi-a-GCST90001806	|| id:ebi-a-GCST90018929	Inverse variance weighted	14	0.023489126	0.02585076	0.36353828
CD27 on unswitched memory B cell || id:ebi-a-GCST90001806	|| id:ebi-a-GCST90018929	Simple mode	14	-0.023317326	0.05016047	0.64972558
CD27 on unswitched memory B cell || id:ebi-a-GCST90001806	|| id:ebi-a-GCST90018929	Weighted mode	14	0.055472694	0.03767882	0.16474595
CD3 on activated CD4 regulatory T cell || id:ebi-a-GCST90001853	|| id:ebi-a-GCST90018929	MR Egger	14	0.020106082	0.0732174	0.78828789
CD3 on activated CD4 regulatory T cell || id:ebi-a-GCST90001853	|| id:ebi-a-GCST90018929	Weighted median	14	0.023543293	0.04164791	0.57187419
CD3 on activated CD4 regulatory T cell || id:ebi-a-GCST90001853	|| id:ebi-a-GCST90018929	Inverse variance weighted	14	-0.046508441	0.03514401	0.18571319
CD3 on activated CD4 regulatory T cell || id:ebi-a-GCST90001853	|| id:ebi-a-GCST90018929	Simple mode	14	-0.020185957	0.07005831	0.7777891
CD3 on activated CD4 regulatory T cell || id:ebi-a-GCST90001853	|| id:ebi-a-GCST90018929	Weighted mode	14	0.011710551	0.04599724	0.80302082

### 3.2 Flow cytometric profiling and diagnostic model construction for indolent PTC

**Figure 1:**
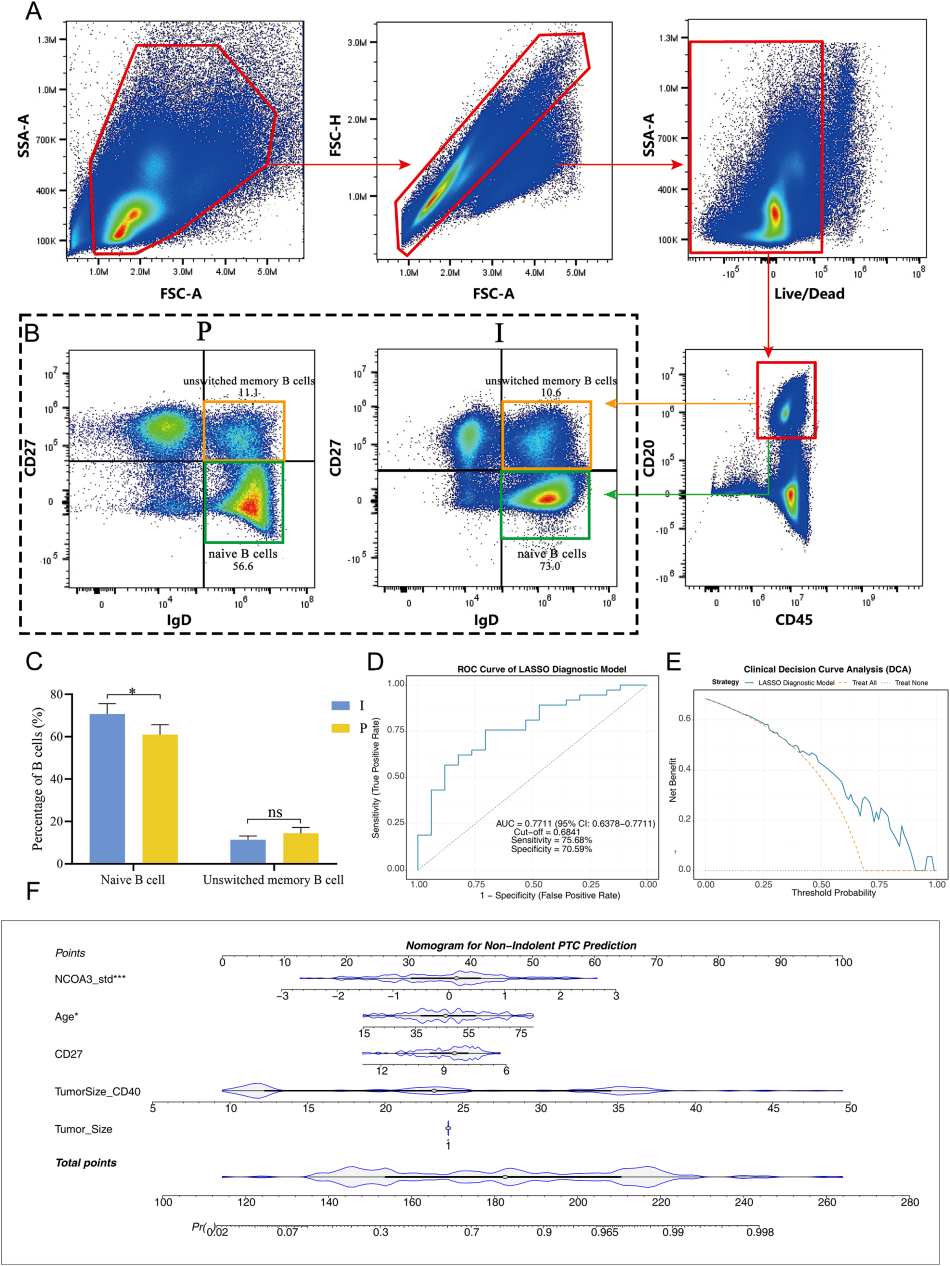
Peripheral blood immune profiling and diagnostic model construction for indolent PTC. **(a)** Gating strategy for flow cytometric analysis of peripheral blood naïve B cells (IgD+CD27-) and unswitched memory B cells (IgD+CD27+) in PTC patients. **(b)** Comparison of peripheral naïve B cell (IgD+CD27-) proportions between indolent and progressive PTC groups. **(c)** Bar chart showing the proportion of peripheral blood B cell subsets between indolent and progressive PTC groups. **(d)** LASSO coefficient profiles for feature selection in the diagnostic model. **(e)** Clinical Decision Curve Analysis (DCA) of the LASSO diagnostic model. **(f)** Receiver Operating Characteristic (ROC) curve and nomogram of the LASSO diagnostic model. * indicates p-value < 0.05, *** indicates p-value < 0.001. PTC, Papillary thyroid carcinoma.

Given that our MR analysis based on blood samples identified naïve B and unswitched memory B cells as potential protective factors against thyroid cancer, we next asked whether these circulating B-cell subsets could help distinguish clinical phenotypes *within* the most common form of the disease. We therefore focused specifically on PTC (>84% of cases (3)) to assess the distribution of these B-cell subpopulations in patients with indolent versus progressive tumors (**Figure 1a**).

Flow cytometric analysis of peripheral blood revealed a significantly higher proportion of naïve B cells (IgD+CD27-) in patients with indolent PTC compared to those with progressive disease (*P* = 0.032). In contrast, the proportion of unswitched memory B cells (IgD+CD27+) did not differ significantly between the two groups (*P* = 0.151) (**Figures 1b, c**).

For high-resolution clinical decision-making, a LASSO regression model was constructed integrating peripheral blood immune indicators (CD27+ unswitched memory B cell proportion) and routine clinical parameters (Age, TumorSize_CD40, Tumor_Size, NCOA3_std) (**Figure 1d**). LASSO regression was employed for feature selection to mitigate overfitting. Receiver operating characteristic (ROC) curve analysis demonstrated that the model exhibited good discriminatory ability, with an area under the curve (AUC) of 0.7711 (95% confidence interval [CI]: 0.6378 - 0.9044, *P* < 0.001. The optimal cut-off value was 0.6841, corresponding to a sensitivity of 75.68% and a specificity of 70.59%. Clinical Decision Curve Analysis (DCA) further validated the model’s clinical utility: within the threshold probability range of 0.1 - 0.8, the LASSO model achieved a higher net benefit than both the “Treat All” and “Treat None” strategies (**Figure 1e**), confirming its capacity to reduce unnecessary interventions while accurately identifying indolent papillary thyroid carcinoma. To facilitate clinical application, a nomogram was developed based on the LASSO model (**Figure 1f**), integrating five core predictive features: NCOA3_std, Age, CD27+ unswitched memory B cell proportion, TumorSize_CD40, and Tumor_Size. Each feature was assigned a weighted score, and the total score (range: 0 - 100) could be converted into an individualized probability of indolent phenotype. The nomogram showed good calibration.

### 3.3 Heterogeneity and functional characterization of tumor-infiltrating B cells in PTC

**Figure 2:**
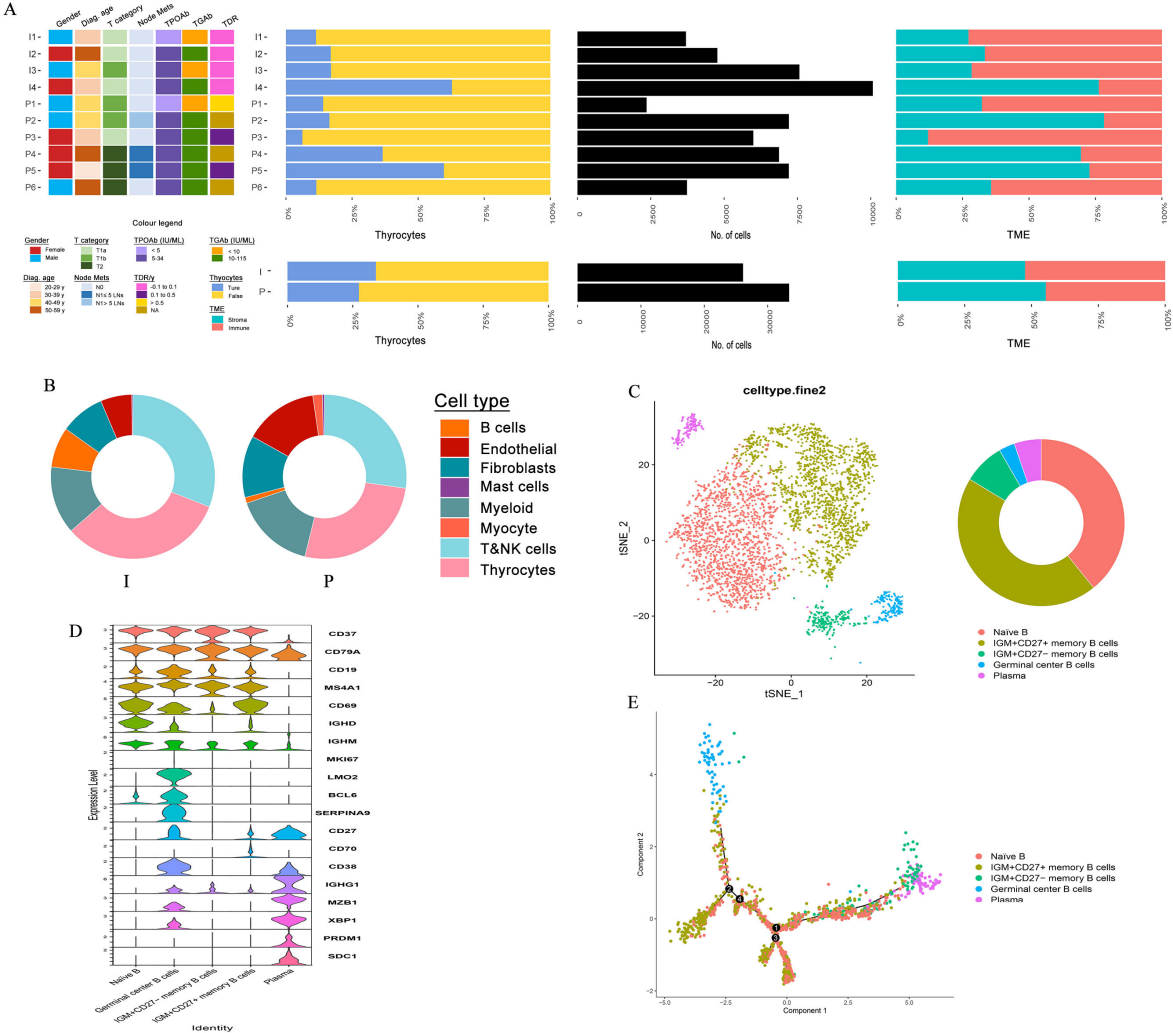
Single-cell transcriptomic analysis reveals heterogeneity and functional characterization of tumor-infiltrating B cells in papillary thyroid carcinoma. **(a)** Clinical information, cell counts, and tumor microenvironment distribution for each sample in the single-cell landscape. **(b)** Cellular composition comparison between indolent (I) and progressive (P) PTC. **(c)** t-SNE visualization of TIL-B subsets from PTCs, color-coded by cell type. The pie chart shows the overall proportions of B cell subset. **(d)** Violin plots showing expression levels of representative marker genes across TIL-B cluster. **(e)** The pseudo-time trajectories of all TIL-B cells. TIL-B cells are ordered along pseudotime trajectories, with the cells color-coded by cell types. TIL-B, Tumor-infiltrating B; TME, tumor microenvironment; PTC, Papillary thyroid carcinoma.

To elucidate the role of tumor-infiltrating B cells (TIL-Bs) in PTC progression, we extended our previous scRNA-seq analysis on 10 early-stage PTC samples, which are identified 8 main cell populations. Analysis of cell proportions revealed significant differences in the TME between indolent and progressive PTC (**Supplementary Figure 5**). Analysis of cell proportions revealed significant differences in the TME between indolent and progressive PTC (**Figure 2a**). The two groups exhibited a fundamental disparity in their cellular composition. Of particular note, a measurable number of B cells were identified in the indolent PTC group, in sharp contrast to their near absence in the progressive group (**Figure 2b**). Upon reclustering B cells, we identified 5 distinct subgroups, naïve B cells (39.2%) and CD27+ memory B cells (44.5%) constitute the predominant B-cell populations among TIL-Bs (**Figures 2c, d**). It is noteworthy that the relative proportions also differed extremely significantly between the two groups: the proportion of naïve B cells (naïve B/TIL-B) was 42.8% vs. 10.8% in the indolent vs. progressive group.

​To further investigate the differentiation potential of naïve B cells within the tumor microenvironment, we reconstructed the pseudotemporal trajectory of TIL-B cells using the reversed graph embedding algorithm in Monocle v.2. The analysis revealed that differentiation from naïve B cells to either CD27+ or CD27- memory B cells was a continuous and gradual transition of transcriptional states, with no evidence of clear branching points or key molecular events dictating lineage commitment towards a specific fate (**Figure 2e**). Gene expression changes progressed smoothly along the pseudotime axis, suggesting substantial plasticity of B cell states or regulation dependent on external signals within the PTC microenvironment. These findings imply that external microenvironmental cues (such as cytokines, T-cell help, or tumor-derived factors) may play a more dominant role in shaping B-cell fate than cell-intrinsic transcriptional programming.

### 3.4 Clinical validation of B-cell protective role

**Figure 3:**
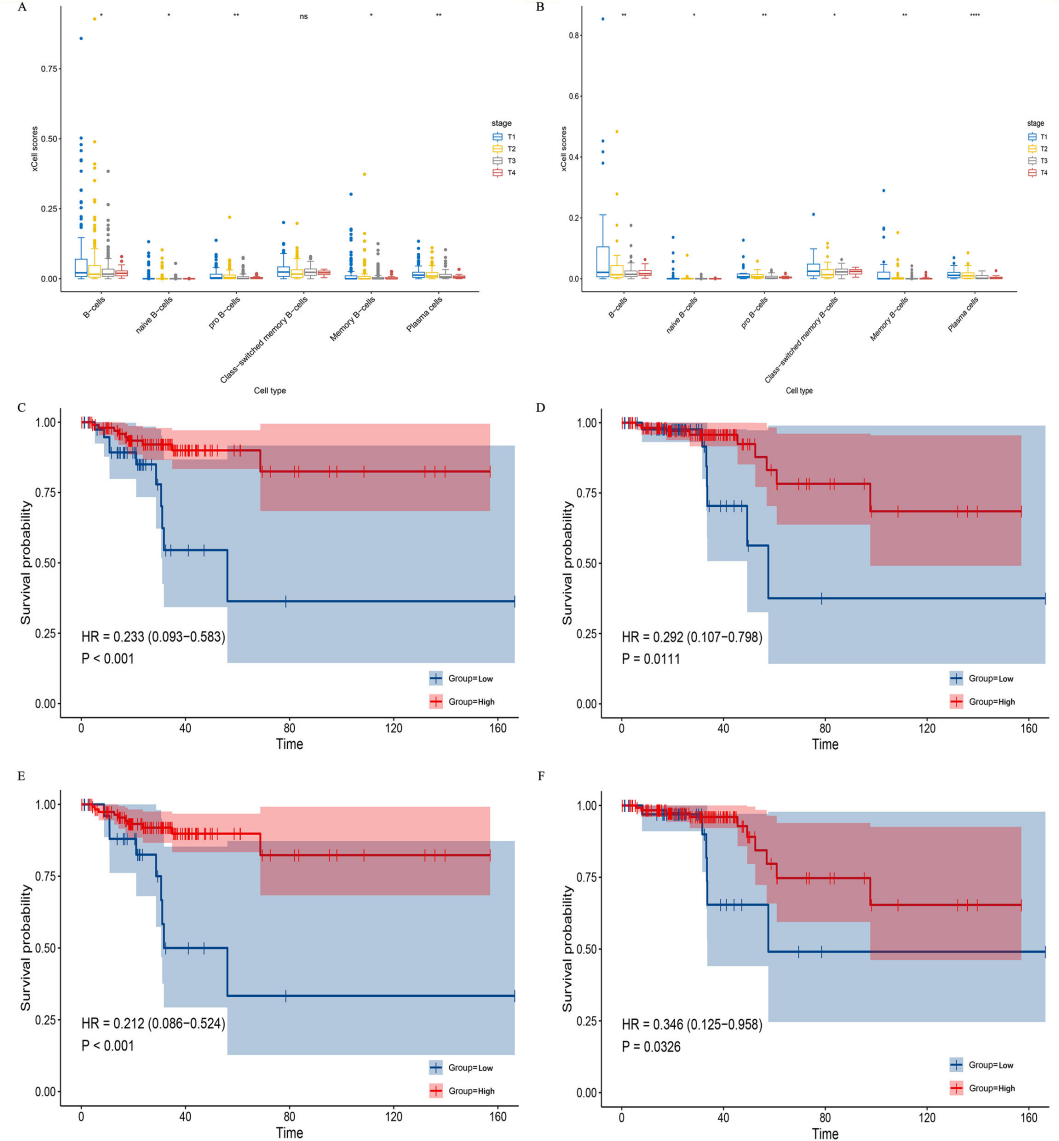
B cell subsets enrichment positively associated with improved T-stage and prognosis in patients with PTC a, **(b)** Box plots comparing the enrichment scores of B cells across different T-stages in PTC patients. Analysis includes all patients **(a)** and patients aged ≥55 years **(b)**. Data were obtained from the TCGA database, and B cell enrichment scores were calculated using the Xcell algorithm. c-f. Kaplan–Meier survival curves depicting DFS and OS for PTC patients aged ≥55 years, stratified by high enrichment scores of specific B cell subsets. Curves show DFS **(c)** and OS **(d)** for patients with high naïve B cell enrichment, and DFS **(e)** and OS **(f)** for patients with high CD27^+^ memory B cell enrichment. Survival differences were assessed using Cox proportional hazards models with log-rank tests. Source note: The PTC data utilized in this figure are from the TCGA database. B cell enrichment scores were computed via the Xcell method based on gene sets identified from our single-cell RNA sequencing analysis and applied to the bulk mRNA-seq data (see Methods). * indicates p-value < 0.05, ** indicates p-value < 0.01, *** indicates p-value < 0.001. DFS, disease-free survival; OS, overall survival; PTC, Papillary thyroid carcinoma; TCGA, The Cancer Genome Atlas.

To independently validate the clinical relevance of the B-cell subsets identified in our study, we analyzed bulk RNA-seq data from the TCGA thyroid carcinoma (THCA) cohort. Using the Xcell algorithm to quantify immune cell infiltration, we observed that high levels of total B-cell infiltration—along with most B-cell subsets (except class-switched memory B cells)—were significantly associated with improved tumor stage (**Figure 3a**). This finding is consistent with our scRNA-seq results and supports a potential protective role of B cells in restraining PTC progression.

Given that advanced age is recognized as a protective factor against tumor progression in active surveillance (AS) management, we further performed a stratified analysis in patients aged ≥55 years. The association between B-cell infiltration and favorable tumor stage remained significant in this subgroup (**Figure 3b**).

We next evaluated the prognostic value of B-cell subpopulations defined by our single-cell sequencing data (**Supplementary Table S4**). Survival analysis in patients ≥55 years, a subgroup at higher risk of adverse outcomes, with patients stratified into high and low enrichment score groups by the cut point of corresponding scores and the low enrichment score group set as the reference group (Ref=1). The results revealed that high enrichment scores for naïve B cells were significantly associated with improved disease-free survival (DFS; HR = 0.233, 95%CI: 0.093-0.583, *P* = <0.001) and overall survival (OS; HR = 0.292, 95%CI: 0.107-0.798, *P* = 0.0111) (**Figures 3c, d**). Similarly, high levels of CD27+ memory B cells correlated with better DFS (HR = 0.212, 95%CI: 0.086-0.524, *P* = <0.001) and OS (HR = 0.346, 95%CI: 0.125- 0.958, *P* = 0.0326) (**Figures 3e, f**).

These results suggest that naïve B cells and CD27+ memory B cells may serve as dual biomarkers capable of both identifying indolent PTC and predicting favorable prognosis, particularly in older patients where clinical decision-making is most challenging. The consistent associations observed across different age groups and clinical endpoints—tumor stage, DFS, and OS—underscore the reliability of these findings.

## 4 Discussion

This integrated exploratory study, incorporating MR, flow cytometry, and multi-omics data, systematically elaborates on the potential causal protective role of specific B-cell subsets in the indolent phenotype of PTC, linking genetic susceptibility to indolent tumor behavior. Building on our prior work that identified tumor-infiltrating B cells as key determinants of PTC indolence (9), we herein novel exploratory genetic evidence that CD20+ IgD+ CD38^-^ naïve B cells and CD27+ unswitched memory B cells in peripheral blood act as independent protective factors against thyroid cancer susceptibility. Notably, the MR findings do not directly model the biological divergence between indolent and progressive PTC; instead, they identify B-cell phenotypes associated with reduced thyroid cancer risk, which are further linked to indolent tumor behavior through complementary experimental approaches. These genetic insights, validated by flow cytometry showing a higher peripheral naïve B-cell proportion in indolent PTC, and corroborated by scRNA-seq revealing these subsets as the predominant intratumoral B-cell populations, collectively suggest a model where a systemic predisposition towards specific B-cell phenotypes may foster a tumor microenvironment conducive to indolence.

Flow cytometric validation confirmed a significantly higher proportion of naïve B cells in the peripheral blood of patients with indolent PTC compared to those with progressive disease. This finding provides clinical corroboration of the genetic inference and establishes proof-of-concept for exploring non-invasive immune monitoring. A critical consideration is the logical link between peripheral blood analyses and the tumor-derived scRNA-seq data, given the profound reshaping capacity of the tumor microenvironment. We must clarify that our study is exploratory in nature. Its primary aim was not to definitively prove equivalence between peripheral and intratumoral B-cell states, but to investigate whether clinically accessible **peripheral immune signatures** might provide clues associated with the functional state of B cells within the TME of indolent PTC. The phenotypic and functional consistency observed across our orthogonal datasets (peripheral MR → peripheral flow cytometry → tumor scRNA-seq) suggests a meaningful association. This may be biologically plausible in the context of indolent PTC, where emerging evidence suggests tumors may actively *recruit* circulating B-cell subsets via chemokine signals, and the TME may lack the extreme immunosuppressive pressures that drive profound phenotypic reprogramming in more aggressive cancers. However, definitive proof of this systemic-to-local link requires future studies employing paired tumor-peripheral single-cell and B-cell receptor sequencing to directly trace clonal relationships and phenotypic evolution.

To address the need for a deeper exploration of protective mechanisms, we integrate our transcriptomic findings with existing literature to propose the following specific, testable hypotheses that synthesize our multi-omics observations (9, 21–24):

1. Enhanced Antigen Presentation as an Initiating Event: Recruited naïve and unswitched memory B cells could act as proficient antigen-presenting cells within the TME. Our scRNA-seq data shows these subsets express key antigen presentation and co-stimulatory molecules (e.g., *HLA* genes, *CD74*, *CD86*). We hypothesize they prime tumor-specific CD4+ T-cell responses, which are critical for sustaining cytotoxic CD8+ T-cell immunity and facilitating the maturation of tertiary lymphoid structures (TLS), a process supported by prior observations in cancer immunity (25). This hypothesis can be tested by *in vitro* co-culture assays measuring tumor antigen-specific CD4+ T cell activation by sorted B-cell subsets.
2. Potentiated B-cell Activation via Modulated Microenvironmental Cues: The enhanced cell-cell interaction network involving *PTPRC* (CD45) and *CD22* identified in our scRNA-seq analysis of indolent PTC may modulate B-cell receptor (BCR) signaling (9). CD22 is a well-known inhibitory coreceptor. We hypothesize that trans-interactions within the TME may counteract CD22-mediated inhibition, thereby lowering the activation threshold for tumor-infiltrating B cells. This premise, consistent with the observed transcriptional profile of proliferation in some intratumoral B cells, can be functionally validated using spatial transcriptomics to confirm ligand-receptor colocalization and by assessing phospho-signaling pathways in B cells isolated from indolent vs. progressive tumors (26).
3. Direct Effector Functions within a Structured Niche: Upon activation and potential differentiation within the TLS niche—a site for which CD27+ memory B cells are known contributors via CXCL13 secretion—B cells may execute direct anti-tumor effector functions (27). We hypothesize this could occur through the secretion of cytotoxic cytokines (e.g., TNF-α) or the local production of tumor-specific antibodies, leading to direct tumor cell killing or antibody-dependent cellular cytotoxicity. This is supported by our prior functional co-culture data (9) and can be further tested by profiling the antibody repertoire from microdissected TLSs and assessing their tumor-neutralizing capacity *in vitro*.

These proposed mechanisms are presented as a cohesive, testable model derived from the correlative patterns in our data. They suggest that a systemic immune landscape enriched in specific B-cell subsets may be permissive for establishing a protective intratumoral environment in indolent PTC, aligning with a model of recruitment and localized activation rather than extensive peripheral reprogramming (28). While the peripheral immune signatures identified are presently insufficient for diagnostic application, they underscore the feasibility of exploring non-invasive biomarkers to infer relevant TME states and generate mechanistic hypotheses for future experimental dissection.

Analysis of the TCGA cohort further reinforced the clinical relevance of these B-cell signatures. High infiltration levels of naïve and CD27+ memory B cells were significantly associated with improved tumor stage and survival outcomes. Notably, this protective effect remained robust in patients aged ≥55 years—a subgroup typically associated with poorer prognosis (29). Against the backdrop of surging thyroid cancer incidence and the limited adoption of AS strategies, specific B-cell subset signatures hold promise as decision-support tools for tailoring surgical or non-surgical management, thereby advancing more precise and individualized AS protocols (30, 31).

Several limitations of our study warrant consideration. First, the GWAS data underlying the MR analyses were primarily derived from European populations, necessitating validation in other ethnic groups. Second, despite comprehensive sensitivity analyses, residual confounding cannot be fully excluded in MR. Third, the specific molecular mechanisms through which these B-cell subsets exert their protective effects remain to be fully elucidated through functional experiments. Finally, the peripheral blood flow cytometry cohort is underpowered for confirmatory conclusions, and all related findings should be considered exploratory proof-of-concept observations that require validation in larger, independent cohorts.

In summary, this exploratory multi-omics study preliminarily establishes a coherent chain of evidence linking genetic susceptibility to clinical manifestations of B-cell–mediated protection in PTC. Its principal contribution lies in delineating future research directions: validating the feasibility of non-invasive monitoring using peripheral B-cell subsets, illuminating the functional plasticity of intratumoral B cells as a basis for mechanistic exploration, and ultimately driving the field toward a higher-resolution understanding of the immune microenvironment in indolent thyroid cancer.

## Data Availability

(1) The data supporting the findings of this study are available from the following sources: Genetic Data for Mendelian Randomization: The summary-level genome-wide association study (GWAS) data for immunophenotypes and thyroid cancer were obtained from the IEU OpenGWAS database (https://gwas.mrcieu.ac.uk/). The specific accession codes are: immunophenotypes (GCST0001391 to GCST0002121) and thyroid cancer (ebi-a-GCST90018929). These are publicly accessible resources. (2) Single-Cell RNA Sequencing Raw Data: The raw sequence data generated in this study have been deposited in the Genome Sequence Archive (GSA) at the National Genomics Data Center, China National Center for Bioinformation/Beijing Institute of Genomics, Chinese Academy of Sciences, under accession number HRA009354. These data are publicly accessible at https://ngdc.cncb.ac.cn/gsa-human. (3) Public Cohort Data: The clinical information and bulk RNA sequencing data for TCGA-Thyroid Carcinoma cohort are publicly available through the Genomic Data Commons (GDC) portal (https://portal.gdc.cancer.gov/).

## References

[1] Parkin DM Bray F Ferlay J Pisani P . Global cancer statistics, 2002. CA Cancer J Clin. (2005) 55:74–108. doi: 10.3322/canjclin.55.2.74, PMID: 15761078

[2] Bray F Laversanne M Sung H Ferlay J Siegel RL Soerjomataram I . Global cancer statistics 2022: GLOBOCAN estimates of incidence and mortality worldwide for 36 cancers in 185 countries. CA Cancer J Clin. (2024) 74:229–63. doi: 10.3322/caac.21834, PMID: 38572751

[3] Boucai L Zafereo M Cabanillas ME . Thyroid Cancer: A Review. JAMA. (2024) 331:425–35. doi: 10.1001/jama.2023.26348, PMID: 38319329

[4] Perrier ND Brierley JD Tuttle RM . Differentiated and anaplastic thyroid carcinoma: Major changes in the American Joint Committee on Cancer eighth edition cancer staging manual. CA Cancer J Clin. (2018) 68:55–63. doi: 10.3322/caac.21439, PMID: 29092098 PMC5766386

[5] Li M Dal Maso L Pizzato M Vaccarella S . Evolving epidemiological patterns of thyroid cancer and estimates of overdiagnosis in 2013–17 in 63 countries worldwide: a population-based study. Lancet Diabetes Endocrinol. (2024) 12:824–36. doi: 10.1016/S2213-8587(24)00223-7, PMID: 39389067

[6] Ringel MD Sosa JA Baloch Z Bischoff L Bloom G Brent GA . 2025 American Thyroid Association Management Guidelines for Adult Patients with Differentiated Thyroid Cancer. Thyroid. (2025) 35:841–985. doi: 10.1177/10507256251363120, PMID: 40844370 PMC13090833

[7] Sugitani I Ito Y Takeuchi D Nakayama H Masaki C Shindo H . Indications and Strategy for Active Surveillance of Adult Low-Risk Papillary Thyroid Microcarcinoma: Consensus Statements from the Japan Association of Endocrine Surgery Task Force on Management for Papillary Thyroid Microcarcinoma. Thyroid. (2021) 31:183–92. doi: 10.1089/thy.2020.0330, PMID: 33023426 PMC7891203

[8] Oh HS Kwon H Song E Jeon MJ Kim TY Lee JH . Tumor Volume Doubling Time in Active Surveillance of Papillary Thyroid Carcinoma. Thyroid. (2019) 29:642–9. doi: 10.1089/thy.2018.0609, PMID: 30864894

[9] Li C Wang P Dong Z Cao W Su Y Zhang J . Single-cell transcriptomics analysis reveals that the tumor-infiltrating B cells determine the indolent fate of papillary thyroid carcinoma. J Exp Clin Cancer Res. (2025) 44:91. doi: 10.1186/s13046-025-03341-7, PMID: 40069827 PMC11895268

[10] Skrivankova VW Richmond RC Woolf BAR Yarmolinsky J Davies NM Swanson SA . Strengthening the Reporting of Observational Studies in Epidemiology Using Mendelian Randomization: The STROBE-MR Statement. JAMA. (2021) 326:1614–21. doi: 10.1001/jama.2021.18236, PMID: 34698778

[11] Larsson SC Butterworth AS Burgess S . Mendelian randomization for cardiovascular diseases: principles and applications. Eur Heart J. (2023) 44:4913–24. doi: 10.1093/eurheartj/ehad736, PMID: 37935836 PMC10719501

[12] Liu W Dong Z Cao W Jin H Li Y Lei P . The Natural Course of Low-Risk Papillary Thyroid Microcarcinoma During Pregnancy: A Prospective Active Surveillance Study. Thyroid. (2025) 35:684–90. doi: 10.1089/thy.2024.0702, PMID: 40402832

[13] Liu W Cao W Dong Z Cheng R . Active surveiilance for low-risk papillary thyroid microcarcinoma: A single center prospective observation study. Chin J Endocrinol Metab. (2022) 38:1068–74. doi: 10.3760/cma.j.cn311282-20220409-00218, PMID: 40668938

[14] Orrù V Steri M Sidore C Marongiu M Serra V Olla S . Author Correction: Complex genetic signatures in immune cells underlie autoimmunity and inform therapy. Nat Genet. (2020) 52:1266. doi: 10.1038/s41588-020-00718-6, PMID: 32948852

[15] Xiao G He Q Liu L Zhang T Zhou M Li X . Causality of genetically determined metabolites on anxiety disorders: a two-sample Mendelian randomization study. J Transl Med. (2022) 20:475. doi: 10.1186/s12967-022-03691-2, PMID: 36266699 PMC9583573

[16] Bowden J Davey Smith G Haycock PC Burgess S . Consistent Estimation in Mendelian Randomization with Some Invalid Instruments Using a Weighted Median Estimator. Genet Epidemiol. (2016) 40:304–14. doi: 10.1002/gepi.21965, PMID: 27061298 PMC4849733

[17] Rogne T Gill D Liew Z Shi X Stensrud VH Nilsen TIL . Mediating Factors in the Association of Maternal Educational Level With Pregnancy Outcomes: A Mendelian Randomization Study. JAMA Netw Open. (2024) 7:e2351166. doi: 10.1001/jamanetworkopen.2023.51166, PMID: 38206626 PMC10784860

[18] Avery L Rotondi N McKnight C Firestone M Smylie J Rotondi M . Unweighted regression models perform better than weighted regression techniques for respondent-driven sampling data: results from a simulation study. BMC Med Res Methodol. (2019) 19:202. doi: 10.1186/s12874-019-0842-5, PMID: 31664912 PMC6819607

[19] Hamilton MG . Maximum likelihood parentage assignment using quantitative genotypes. Heredity (Edinb). (2021) 126:884–95. doi: 10.1038/s41437-021-00421-0, PMID: 33692533 PMC8178362

[20] Li Y Lu F Yin Y . Applying logistic LASSO regression for the diagnosis of atypical Crohn’s disease. Sci Rep. (2022) 12:11340. doi: 10.1038/s41598-022-15609-5, PMID: 35790774 PMC9256608

[21] Fang X Huang X Lu J Su D . Causal role of immune cells in thyroid cancer: a bidirectional Mendelian randomization study. Front Immunol. (2024) 15:1425873. doi: 10.3389/fimmu.2024.1425873, PMID: 38953025 PMC11215042

[22] Li YY Li SJ Liu MC Chen Z Li L Shen F . B cells and tertiary lymphoid structures are associated with survival in papillary thyroid cancer. J Endocrinol Invest. (2023) 46:2247–56. doi: 10.1007/s40618-023-02072-w, PMID: 37004696

[23] Zhang W Zhang Y Liu Z Wang Z Wang H Ji X . PROS1-MERTK Axis Drives Tumor Microenvironment Crosstalk and Progression in Papillary Thyroid Microcarcinoma. Adv Sci (Weinh). (2025) 12:e13474. doi: 10.1002/advs.202413474, PMID: 40433916 PMC12376618

[24] Wang Z Ji X Zhang Y Yang F Su H Zhang H . Interactions between LAMP3+ dendritic cells and T-cell subpopulations promote immune evasion in papillary thyroid carcinoma. J Immunother Cancer. (2024) 12:e008983. doi: 10.1136/jitc-2024-008983, PMID: 38816233 PMC11141193

[25] Li Y Bhargava R Tran JT Blane TR Peng L Luan F . Blocking plasma cell fate enhances antigen-specific presentation by B cells to boost anti-tumor immunity. Nat Commun. (2025) 16:4454. doi: 10.1038/s41467-025-59622-4, PMID: 40360528 PMC12075458

[26] Liao T Zeng Y Xu W Shi X Shen C Du Y . A spatially resolved transcriptome landscape during thyroid cancer progression. Cell Rep Med. (2025) 6:102043. doi: 10.1016/j.xcrm.2025.102043, PMID: 40157360 PMC12047530

[27] Fridman WH Meylan M Petitprez F Sun CM Italiano A Sautès-Fridman C . B cells and tertiary lymphoid structures as determinants of tumour immune contexture and clinical outcome. Nat Rev Clin Oncol. (2022) 19:441–57. doi: 10.1038/s41571-022-00619-z, PMID: 35365796

[28] Ao W Chen S Liu T Wang B Zhao W . Genomic Characterization of Papillary Thyroid Carcinoma: Age Differences in Tumor Aggressiveness and Immune Infiltration. Diagnostics (Basel). (2025) 15:2937. doi: 10.3390/diagnostics15232937, PMID: 41374320 PMC12691218

[29] Xing M Lin S Mathur A Li Y Bendlova B Kuklikova V . Genetic modification of the AJCC classification of papillary thyroid cancer: an international, multicentre, retrospective cohort study. Lancet Oncol. (2025) 26:1382–92. doi: 10.1016/S1470-2045(25)00399-7, PMID: 41038186 PMC12969324

[30] Liu W Cao W Dong Z Cheng R . Can Active Surveillance Management be Developed for Patients With Low-Risk Papillary Thyroid Microcarcinoma? A Preliminary Investigation in a Chinese Population. Endocr Pract. (2022) 28:391–7. doi: 10.1016/j.eprac.2022.01.013, PMID: 35124241

[31] Zhu Q Liu J Hu J Zhang Y . The Epidemiological Landscape of Thyroid Cancer and Estimates of Overdiagnosis in China: A Population-Based Study. Thyroid. (2025) 35:307–20. doi: 10.1089/thy.2024.0583, PMID: 39970038

