# Supplementary material for: Unraveling the indolence of papillary thyroid carcinoma: an exploratory study on B-cell subsets based on genetic predisposition and tumor immunity
Source: Front Immunol. 2026 Mar 18;17:1769020. doi: 10.3389/fimmu.2026.1769020 (PMC13038570; doi:10.3389/fimmu.2026.1769020)
Supplement: Supplementary file 1 [file DataSheet1.zip › Supplementary_Figurelegends.docx]

Supplementary Material

## Supplementary Figures

**Supplementary Fig. S1:** Scatter plots of the causal effects between the 17 exposure factors and TC based on the IVW method. The horizontal axis represents the effect of SNPs on the exposure factors, whereas the vertical axis represents their effect on the outcome. Positive slopes indicate risk factors (OR > 1), while negative slopes indicate protective factors (OR < 1).

**Supplementary Fig. S2:** Forest plot showing the causal effect of each SNP on TC risk.

**Supplementary Fig. S3:** Funnel plots used to assess the overall heterogeneity of the MR estimates for the influence of the 17 exposure factors on TC.

**Supplementary Fig. S4:** Leave-one-out plots used to visualize the causal effects of the 17 exposure factors on TC risk, with one SNP excluded at a time.

**Supplementary Fig. S5: scRNA-seq reveals the cellular heterogeneity of early-stage PTC.**t-SNE visualization of 74,714 cells from PTC tumors, color-coded by the 8 major cell types identified through unsupervised clustering.
